# Neither uni- nor multi-modal exercise interventions improved single- and dual-task gait performance in physically active healthy elderly – a pilot study

**DOI:** 10.1186/s12877-025-06537-w

**Published:** 2025-11-04

**Authors:** Constantin W. Freitag, Martin Behrens, Robert Bielitzki, Tom Behrendt, Khaldoon O. Al-Nosairy, Francie H. Stolle, Gokulraj T. Prabhakaran, Rosalie Beyer, Cynthia Moffack Djuloun, Hagen Thieme, Michael B. Hoffmann, Lutz Schega

**Affiliations:** 1 Department of Sport Science, Institute III, Otto von Guericke University Magdeburg, Zschokkestraße 32, Magdeburg, 39104 Germany; 2 University of Applied Sciences for Sport and Management Potsdam, Olympischer Weg 7, Potsdam, 14471 Germany; 3 Department of Orthopaedics, Rostock University Medical Center, Rostock, 18057 Germany; 4 Ophthalmic Department, Section for Clinical and Experimental Sensory Physiology, University Hospital Magdeburg, Leipziger Str. 44, Magdeburg, 39120 Germany; 5 Center for Behavioral Brain Research, Magdeburg, 39106 Germany

**Keywords:** Aging, Resistance training, Motor-cognitive training, Gait, Overground walking

## Abstract

**Purpose:**

Aging is an inevitable process leading, inter alia, to the loss of muscle mass as well as the decrease in physical and cognitive function. These age-related impairments translate into a reduced gait performance and an increased risk of falls, which can be tackled with resistance training, Unimodal intervention (UMI). However, Multimodal intervention (MMI), i.e. combined motor-cognitive and resistance training, might be a more promising approach to increase physical and cognitive function in old adults. Therefore, this pilot study aimed to investigate the effects of MMI, compared to UMI, on gait and cognitive performance in elderly participants. We hypothesized that MMI will increase gait and cognitive performance to a larger extent than UMI.

**Methods:**

In this two-arm randomized controlled pilot study, 24 healthy active elderly participants completed the MMI (12 participants, 72.6 ± 5.4 years) and the UMI (12 participants, 70.4 ± 5.3 years). Both groups trained for 12 weeks, two times a week for 60 min, respectively. MMI consisted of motor-cognitive training directly followed by resistance training, while UMI consisted of a stand-alone resistance training. Three weeks before and after the interventions, gait performance (e.g., stride length, velocity, minimum toe clearance) was assessed during single- and dual-task walking trials using inertial measurement units. During dual-task walking, participants walked and concurrently performed different cognitive tasks in a random order: (i) reaction time task, (ii) N-back-task, and (iii) letter fluency task with two difficulty levels, respectively. Data were analyzed with repeated measures analyses of covariance (Time×Intervention×Condition).

**Results:**

Although the analyses of the progression of the external load used during resistance training showed a significant increase over the training period (e.g. leg press *p* < 0.001,$\:{{\upeta\:}}_{\mathrm{p}}^{2}$=0.678), there was no improvement of gait or cognitive performance in active old adults after neither MMI nor UMI.

**Conclusion:**

Against our hypothesis, the present pilot study indicated that neither a 12-week MMI nor UMI seems to have a sizable impact on gait parameters and cognitive performance in physically active healthy adults. Still, a significant increase in the external load used during resistance training was observed, implying neuromuscular adaptations, which, however, did not translate into a higher gait and/or cognitive performance.

**Clinical trial number:**

German Clinical Trial Register ID: DRKS00022519/05.08.2020

**Supplementary Information:**

The online version contains supplementary material available at https://doi.org/10.1186/s12877-025-06537-w .

## Introduction

Aging is a continuous process resulting in, inter alia, skeletal muscle atrophy (sarcopenia) [1] and a reduction in neural drive to the muscles finally causing a decline in maximal and explosive muscle strength (dynapenia) [2, 3] and function. Moreover, aging might impair cognitive functions including decreased executive functions [4]. Both the age-related decline in physical and cognitive function can affect daily activities by deteriorating gait performance and increasing the risk of falls [5, 6], a major cause of injuries in the elderly [7].

Several spatiotemporal gait parameters, such as stride length, gait velocity, and gait variability measures have been shown to be predictive for the risk of falls [8, 9]. In particular, the minimum toe clearance (MTC) appears to be a promising marker for evaluating walking performance and perhaps the risk of falling in older adults [10]. The MTC describes the smallest distance between the ground and toe during the mid-swing phase of the gait cycle [8] and the lower the MTC and the larger its variability, the higher the risk of sustaining a fall [10]. Gait performance, i.e., motor function and control during walking, is often evaluated during motor-cognitive dual-task walking [11–16], which is characterized by performing a concurrent cognitive task during walking and often results in increased gait variability measures [8, 17]. The worsening of gait performance might be attributable to the reduced processing capacity for the motor task (central capacity sharing model) and/or the sequential neural processing of the motor and cognitive interference task (bottleneck model) [18]. Consequently, performance in at least one task diminishes, e.g., gait performance, a phenomenon known as dual-task costs (DTC). This is of particular importance, given that daily activities often require multitasking, such as walking while thinking, texting, or phoning. Importantly, a lower motor-cognitive dual-task walking performance, i.e., higher DTC, is related to a higher risk of falling [19].

Since falls can lead to fear of future falls [20], a loop of physical inactivity might begin promoting sarcopenia, dynapenia, as well as cognitive dysfunction [21], and thus, increasing the risk of falling. To counteract this, resistance training promises a suitable interventional strategy to increase neuromuscular function and functional performance in older adults [6]. In this regard, resistance training has been shown to increase postural control and gait performance in elderly [22]. Furthermore, resistance training might also have positive effects on cognitive performance due to functional (e.g., in the frontal lobe) and structural brain adaptations (e.g., lower white matter atrophy and smaller white matter lesion volumes) [23].

Further, there is evidence that motor-cognitive dual-task training (i.e., the concurrent execution of motor and cognitive tasks) [24] elicits structural and functional changes in the aging brain, which were associated with an improved cognitive performance [25–28]. Although the evidence is inconclusive, concurrent training of motor and cognitive tasks might be a more promising approach for enhancing cognitive performance compared with single motor or cognitive training [13, 29]. Given that both, the age-related decline in strength and cognitive function, have an impact on gait performance, a multimodal intervention (MMI) combining resistance training and motor-cognitive dual-task training [24] could be more effective in improving gait performance (e.g., stride length, gait velocity, MTC and their variability) than resistance training alone. This is of relevance, since these gait measures [8–10], especially recorded during dual-task walking [19], have been shown to be predictive for the risk of falls. Therefore, improving gait performance with MMI might be a promising approach for fall prevention in older adults.

As far as the authors of the present study are aware, there is no experimental trial that has investigated the effect of a stand-alone resistance training compared to a motor-cognitive dual-task training directly followed by a resistance training intervention on gait and cognitive performance in healthy elderly. Therefore, the present study compared the influence of a 12-week MMI (motor-cognitive dual-task training + resistance training) and a unimodal intervention (UMI, stand-alone resistance training, twice a week, lasting 60 min each, 24 training sessions in total) on gait performance and cognitive performance in healthy old adults. For that purpose, gait parameters (i.e., stride length, gait velocity, MTC), and their respective coefficient of variation (CoV) [8] were recorded during single-task and motor-cognitive dual-task walking before and after the training intervention. Additionally, the DTC (relative changes between single-task and dual-task performance) were calculated to assess the cognitive demands during walking [30].

It was hypothesized that the MMI leads to higher improvements in gait performance, especially during dual-task walking, and cognitive performance compared to UMI. Furthermore, a decrease in DTC was expected after the MMI.

## Methods

### Study design

This two-arm randomized controlled pilot study was conducted from August 2020 to December 2022. The interventions and measurements were performed in the laboratories of the Sport Science Department at the Otto von Guericke University Magdeburg. The study was carried out in accordance with the Declaration of Helsinki and approved by the Ethics Committee of the University Medical Faculty Magdeburg (32/18). Reporting was performed in accordance with the Consolidated Standards of Reporting Trials (CONSORT) Statement for randomized pilot trials [31]. The data presented in this article are part of a larger study investigating the effects of a MMI versus UMI on visual, motor, and cognitive performance as well as structural and functional brain adaptations in glaucoma patients and healthy controls (German Clinical Trial Register, ID: DRKS00022519/05.08.2020, https://drks.de/search/de/trial/DRKS00022519). All participants signed the informed consent form before participating in this study.

### Participants

**Fig. 1:**
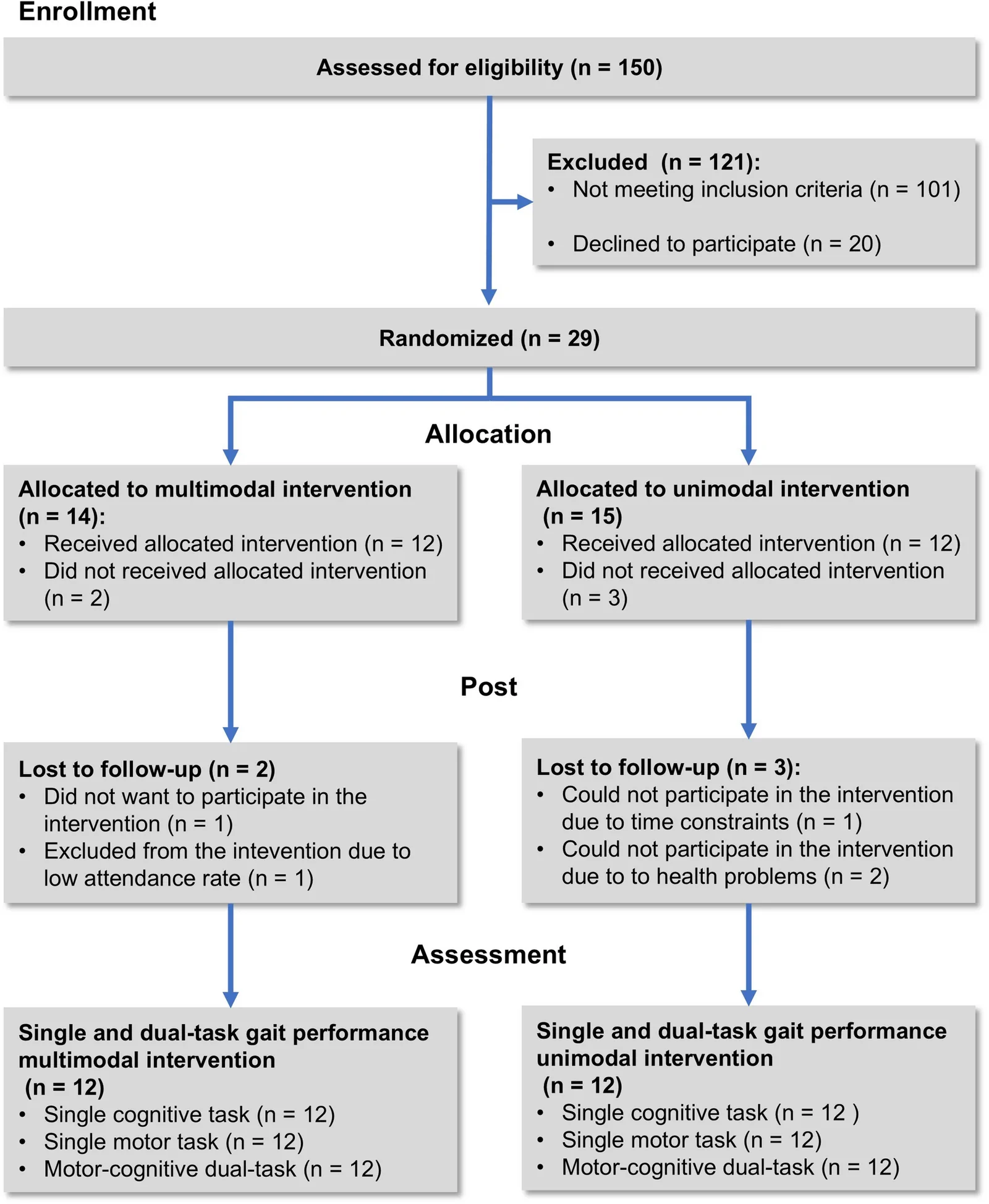
Study schedule of enrollment, intervention, and assessments

As mentioned above, the present data are secondary outcomes of a larger randomized controlled trial focusing on functional brain connectivity as the primary outcome. Therefore, the sample size is based on the study by Demiracka and colleagues [32], who reported significant effects of the Life Kinetik^®^ intervention (*n* = 21) on functional brain connectivity compared to a control group (*n* = 11). It should be noted that the data-acquisition/funding epoch coincided with the COVID-19 pandemic, such that the total of 24 participants was the maximum possible. Recruitment process was conducted at the Department of Ophthalmology at the University Hospital Magdeburg via the local newspaper. Participants were eligible when they met the following inclusion criteria: (i) age ≥ 60 and (ii) the ability to walk at least six minutes without support. Exclusion criteria were defined as follows: (i) eye diseases/surgeries affecting visual function, (ii) neurological disorders, (iii) rheumatism, (iv) cardiovascular disorders, (v) stroke, (vi) orthopedic diseases including arthrosis (grade II or higher), musculoskeletal impairment, tendinitis, tenosynovitis, myositis, prosthesis in the lower extremities, and joint replacements. The allocation was performed at the Department of Sport Science. Participants gave their informed consent to voluntarily participate in the present study and were randomly assigned to either the MMI or the UMI using counterbalanced randomization (allocation ratio was 1:1) by a computer-generated table of random numbers (Fig. 1). For participant characteristics see results.

### General procedure

All outcomes were assessed during a period of three weeks before and after the interventions, respectively. The assessors of the measurements were not blinded to the group allocation because they also supervised the interventions.

At the beginning of the measurements, participants signed the informed consent and were given the Freiburger Questionnaire on Physical Activity [33] to assess the level of physical activity. Furthermore, age, height, and weight of each participant were recorded. The complete testing procedure including the methods for recording and calculating the spatiotemporal gait parameters have been described in detail by Freitag et al. [30]. Briefly, over two consecutive days, participants performed single-tasks (at the beginning of each day) and three dual-task walking tasks at individuals’ comfort velocity over a 10 m track back and forth for 180 s, respectively. The dual-task walking tasks were conducted in a random order and in this regard, one was performed on day one and the other on day two.

During dual-task walking, the participants performed three cognitive tasks: (i) reaction time task, (ii) N-back-task, and (iii) letter fluency task with two levels of difficulty, respectively. Additionally, all cognitive tasks were performed as a separate single-task to calculate DTC for cognitive performance. DTC for gait performance were calculated using the single-task and dual-task walking performance.

Spatiotemporal gait parameters (stride length, gait velocity, MTC) and their respective CoV (CoV = 100 x standard deviation/mean) were assessed using inertial measurement units (XSENS MTW Awinda, Movella, Delft, Netherlands; sampling frequency 100 Hz) placed proximal on each foot and the sternum. The gait parameters were calculated using the algorithm developed by Hamacher et al.^8^. Due to the corona virus pandemic, participants were advised to wear a FFP2 face mask.

### Intervention

Both intervention groups completed a 12-week training program with two sessions per week (i.e., a total of 24 sessions) on non-consecutive days. Each exercise session lasted 60 min and was guided by experienced instructors.

The MMI was split into the following sequences: (i) motor-cognitive training based on the Life Kinetik^®^ program [34] and (ii) resistance training. The Life Kinetik^®^ program consisted of simultaneously performed motor-cognitive dual-tasks. The progression of the exercises followed a standardized schedule. They were designed to be complex and intense enough to ensure that successful completion is initially unattainable. If the exercises were performed correctly in 6 out of 10 trials, the instructor introduced a more advanced variation of the exercise [34]. The training sessions included exercises such as: (i) balls with different colors (e.g., yellow, green, red) were thrown in a circle, whereby a corresponding name must be said for each color (e.g., yellow = persons own name, green = name of the person to whom the ball has to be thrown. (ii) Participants stand next to each other. After an announcement (e.g., left, right, front, back) the participants walked in the corresponding direction (line of vision remained the same, i.e., no turning of the body), whereby the corresponding name for each direction was varied (e.g., right = 1, left = 2, front = 3, back = 4). The duration of the motor-cognitive training and the resistance training varied from month to month (Table 1). The duration was increased and the exercises changed every month with weekly variations from the beginning of the 2th month resulting in a progressive reduction in the time for the resistance training.

**Table 1:** MMI training schedule

Training components	Months		
	1 st Month	2nd Month	3th Month
Life Kinetik^®^	10 min	20 min	30 min
Resistance Training	40 min	30 min	20 min
Cool Down	10 min	10 min	10 min

The UMI consisted of nine exercises. Prior to the main exercises, a 10-minute standardized warmup was performed containing fast walking and dynamic stretching of the upper and lower extremities. The resistance exercises were performed using free weights and exercise machines in a fixed order. Subjects had to perform 2 sets with 20 repetitions of each exercise using an external load (i.e., weight) that corresponded to a moderate to somewhat severe (3–4) rating on a perceived exertion (RPE) scale (Borg scale, 1–10) [35]. The exercises were metronome paced at a cadence of 30 bpm (i.e., 2 s concentric/2 s eccentric).

Both interventions were completed with a cool down of 10-min static stretching for the major muscle groups (e.g., standing side stretch, standing forward fold, overhead triceps stretch).

Due to the fact that this intervention was part of a larger study investigating the effects of a MMI versus UMI on visual, motor, and cognitive performance as well as structural and functional brain adaptations in glaucoma patients, all exercises were performed while sitting or standing because exercising in a supine position affects the intraocular pressure [36, 37], which represents a major risk factor of open angle glaucoma [38]. From the second month of the intervention period, both intervention groups trained with two training plans that were performed alternately. A detailed overview of the training program with exercise variables is provided in the Table 2. The participants were allowed to continue with their usual physical activities.

**Table 2:** Overview of the resistance training for the unimodal intervention and multimodal intervention throughout the intervention period. Letter in brackets indicate the major muscles involved in the exercise according to Haff and triplett (2016) [39]

Week		Unimodal Intervention	Multimodal Intervention	Major Muscles Involved
Weeks 1–4		1. Seated leg press (A) 2. Lever Pulldown (B) 3. Lever seated twist (C) 4. Lever chest press (D) 5. Cable curl (E) 6. Lever back extension (F) 7. Cable pushdown (G) 8. Cable upright row (H) 9. Seated leg curl (I)	1. Seated leg press (A) 2. Lever Pulldown (B) 3. Lever seated twist (C) 4. Lever chest press (D) 5. Cable curl (E) 6. Lever back extension (F) 7. Cable pushdown (G) 8. Cable upright row (H) 9. Seated leg curl (I)	A. Gluteus maximus, Semimembranosus, Semitendinosus, Biceps femoris, Vastus lateralis, Vastus intermedius, Vastus medialis, Rectus femoris B. Latissimus dorsi, Teres major, Middle trapezius, Rhomboids, Posterior deltoids C. External abdominal oblique, Internal abdominal oblique D. Pectoralis major, Anterior deltoids, Triceps brachii E. Biceps brachii, Brachialis, Brachioradialis F. Erector spinae G. Triceps brachii H. Deltoids, Upper trapezius I. Semimembranosus, Semitendinosus, Biceps femoris J. Vastus lateralis, Vastus intermedius, Vastus medialis, Rectus femoris K. Deltoids L. Latissimus dorsi, Triceps brachii
Weeks 5–8	Day 1	1. Seated leg press (A) 2. Cable straight back seated row (B) 3. Lever seated twist (C) 4. Lever chest press (D) 5. Cable curl (E) 6. Lever back extension (F) 7. Cable pushdown (G) 8. Cable upright row (H) 9. Lever leg extension (J)	1. Seated leg press (A) 2. Cable straight back seated row (B) 3. Lever seated twist (C) 4. Lever pec dec fly (D) 5. Cable upright row (H) 6. Cable curl (E)	
	Day 2	1. Seated leg press (A) 2. Lever pulldown (B) 3. Lever seated twist (C) 4. Lever pec dec fly (D) 5. Reverse cable curl (E) 6. Lever back extension (F) 7. Cable pushdown (G) 8. Dumbell seated lateral raise (K) 9. Seated leg curl (I)	1. Cable pushdown (G) 2. Lever pulldown (B) 3. Lever back extension (F) 4. Lever chest press (D) 5. Dumbbell seated lateral raise (K) 6. Seated leg curl (I)	
Weeks 9–12	Day 1	1. Seated leg press (A) 2. Cable straight back seated row (B) 3. Lever chest press (D) 4. Lever seated twist (C) 5. Cable Curl (E) 6. Dumbbell seated front raise (K) 7. Seated leg curl (I) 8. Lever back extension (F) 9. Cable pushdown (G)	1. Seated leg press (A) 2. Dumbbell seated front raise (H) 3. Reverse cable curl (E) 4. Lever chest press (D) 5. Lever seated twist (C)	
	Day 2	1. Seated leg press (A) 2. Cable seated straight arm pulldown (L) 3. Lever seated twist (C) 4. Lever pec dec fly (D) 5. Reverse cable curl (E) 6. Seated triceps press (G) 7. Dumbbell seated lateral raise (K) 8. Lever leg extension (J) 9. Lever back extension (F)	1. Seated leg curl (I) 2. Lever pec dec fly (D) 3. Lever back extension (F) 4. Seated triceps press (G) 5. Cable seated straight arm pulldown (L)	

### Motor and cognitive dual-task costs

DTC were calculated as follows:

1 $$\:\begin{array}{c}DTC=\frac{Single\:task-Dual\:task}{Single\:task}\times\:100\end{array}$$

2 $$\:\begin{array}{c}DTC=\frac{Dual\:task-Single\:task}{Dual\:task}\times\:100\end{array}$$

Single task and Dual task are the single- and dual-task performance, respectively. Equation (1) was used when a higher measurement value reflected a better performance. Otherwise, when a lower measurement value reflected a better performance, equation (2) was used. Hence, positive values indicate higher DTC, while negative values reflect a better Dual task performance compared with the Single task performance.

### Statistical analysis

Data were analyzed using the JASP Statistics (Version 0.19.3.0, University of Amsterdam, Amsterdam, Netherlands). Differences between groups in the anthropometric data were checked, depending on distribution of normality, with independent t-tests or Mann-Whitney test. Since previous studies have shown that repeated measures analysis of covariance (ANCOVA) to be robust against moderate violation of normality [40], nonparametric tests were not used to check for differences. Consequently, a three-way repeated measures ANCOVA with the factors INTERVENTION (MMI vs. UMI), CONDITION (single- vs. dual-task), and TIME (pre vs. post) was conducted. Due to difference in distribution of sex between groups it was used as covariate. If a violation of sphericity was detected, the Greenhouse-Geisser correction was applied. To interpret the results and to clarify the practical/clinical relevance of interventional studies it is advisable to use effect sizes [41]. Thus, the effect size partial-eta squared ($\:{{\upeta\:}}_{\mathrm{p}}^{2}$) as well as Cohen’s d (d) were calculated and interpreted according to Lakens [41]: small ($\:{{\upeta\:}}_{\mathrm{p}}^{2}$ ≥ 0.01, d ≥ 0.2), medium ($\:{{\upeta\:}}_{\mathrm{p}}^{2}$ ≥ 0.06, d ≥ 0.5), and large ($\:{{\upeta\:}}_{\mathrm{p}}^{2}$ ≥ 0.14, d ≥ 0.8). In case of TIME x INTERVENTION x CONITION or TIME x INTERVENTION effects as well main effects of TIME, Bonferroni-corrected post-hoc tests were performed. In case of large main effects, the results were interpreted as relevant if $\:{{\upeta\:}}_{\mathrm{p}}^{2}$ ≥ 0.14 or d ≥ 0.8. Due to pilot character of the present study, a sample size calculation, based on the observed effect sizes, was conducted (G*Power 3.1.9.7, Heinrich Heine University Düsseldorf, Düsseldorf, Germany) to provide data on sufficient sample sizes for future randomized controlled trials. In addition, a two-way repeated ANOVA was performed with the factors INTERVENTION (MMI vs. UMI) and TIME (first, second, and third month) to analyze the progression of the external load during the resistance training (i.e., leg press, seated leg curls) as an indirect marker of neuromuscular adaptations, which could have an impact especially on the spatiotemporal gait parameters. The external load per exercise was averaged over each month. 

## Results

### Characteristics of participants

In total, 29 healthy participants were recruited. Fourteen participants (8 females, age: 71.9 ± 5.4 years, height: 164.1 ± 8.5 cm, body mass: 72.3 ± 22.6 kg) received the MMI and 15 participants (9 females, age: 70.3 ± 5.0 years, height: 170.7 ± 9.9 cm, body mass: 78.1 ± 11.9 kg) received the UMI. The Freiburger Questionnaire on Physical Activity [33] indicated that overall physical activity was on average 10.8 h for the MMI group and 15.7 h per week for the UMI group. All participants had an attendance rate of at least 80%. Due to dropouts only data from 24 participants (12 MMI, 12 UMI) were included in the final analysis. Due to processing issues regarding the MTC and the MTC_CoV_, only 23 participants (11 MMI, 12 UMI) were included in the final analysis. Furthermore, due to technical issues during the single-task walking trial at baseline measurement only 21 participants (11 MMI, 10 UMI) were included into the final DTC analysis. Moreover, due to an extreme outlier during the N-Back task at the baseline measurement only 23 participants (12 MMI, 11 UMI) were included into the final (DTC) cognitive performance analysis. The results of the sample size calculations are presented in supplementary Table A and B. An overview of all parameters including the p-values and effect sizes is presented in supplementary Tables C-E).

### Outcome measures

No relevant interactions and main effects were found for gait velocity, stride length, MTC, and their respective CoVs (Supplementary table C) as well as for cognitive performance (Supplementary table E). (Supplementary table D).

The analysis of the external load progression showed a TIME x INTERVENTION interaction for leg press (F(1.253,27.569) = 6.219, *p* = 0.014$\:{{\upeta\:}}_{\mathrm{p}}^{2}$ = 0.221). Post-hoc analysis showed a significant higher external load for the UMI compared to the MMI during the 2nd month (*p* = 0.023, d = 0.955, mean difference = 15.667 kg, 95% CI = 2.329–29.004%, 95% CI for d = 0.420–2.330) and the 3rd month (*p* = 0.014, d = 1.270, mean difference = 20.833 kg, 95% CI = 4.695–36.972%, 95% CI for d = 0.414–2.954).

Further, TIME effects were detected for leg press (F(1.253.27.569) = 46.361, *p* < 0.001, $\:{{\upeta\:}}_{\mathrm{p}}^{2}$ = 0.678) and seated leg curls (F(1.305,28.705) = 21.194, *p* < 0.001, $\:{{\upeta\:}}_{\mathrm{p}}^{2}$ = 0.491). Post-hoc analysis showed a significant increase in external load over the three months for the leg press: 1 st and 2nd month (*p* < 0.001, d = 0.498, mean difference = −8.167 kg, 95% CI = −11.136 - −5.197, 95% CI for d = 0.764 − 0.232), 2nd and 3rd month (*p* < 0.001, d = 0.254, mean difference = −4.167 kg, 95% CI = −6.492 - −1.841%, 95% CI for d = 0.427 − 0.081). Furthermore, the post-hoc analysis showed an increase in external load between the 1 st to 2nd months for the leg curl (*p* < 0.001, d = −0.436, mean difference = −2.729 kg, 95% CI = −4.296 - −1.163%, 95% CI for d = 0.739 − 0.133) and 2nd to 3rd month (*p* = 0.037, d = 0.147, mean difference = −0.917 kg, 95% CI = −1.788 - −0.046, 95% CI for d = 0.297 − 0.004).

## Discussion

The present pilot-study investigated the effect of a 12-week MMI versus UMI on spatiotemporal gait parameters recorded during single- and dual-task walking as well as cognitive performance in healthy older adults. Contrary to our hypothesis, both training interventions failed to improve gait parameters, their respective CoVs, cognitive performance as well as motor and cognitive DTC. Nevertheless, both interventions provoked progress regarding the external load during the training period indicating increased strength capabilities due to training-induced neuromuscular adaptations.

The results of the present study revealed that none of the two interventions positively influenced gait or cognitive performance. This finding is surprising given that positive intervention effects on the dependent variables (e.g., stride length, gait velocity, MTC and their variability) were expected for both the UMI and MMI in healthy elderly. To the knowledge of the authors, no previous study has investigated the influence of a comparable MMI on gait performance, cognitive performance, as well as motor and cognitive DTC in healthy elderly participants. However, Wollesen et al. [42] showed that a progressive stand-alone resistance training and dual-task training (e.g. fast walking in combination with visual and balance tasks, 12 sessions, 60 min, 12 weeks) led to an improved gait performance in healthy elderly participants (i.e., increased step length). Furthermore, Singh et al. [43] demonstrated that a resistance training (2–3 days/week, 75 min, 24 weeks) increased cognitive performance in patients with mild cognitive impairment. Moreover, Castano et al. [44] showed that combining resistance training with a verbal fluency task (2 days/week, 60 min, 16 weeks) enhanced plasma brain-derived neurotrophic factor level, which is associated with cognitive performance, compared to traditional resistance training in healthy elderly participants.

It could be assumed that the external load used during resistance training, which was prescribed based on the RPE, might have been too low to induce neuromuscular adaptations translating in an improvement in gait performance [6]. In this regard, it should also be considered that both intervention groups had a high physical activity (10.8 h/week and 15.7 h/week, respectively), which is above the average compared to the same age (≥ 70 years, 9.9 h/per week [33]). On top of that, ST gait velocity of participants of the present study (1.26 m/s) was above the average of elderly without gait impairments (1.05 m/s) [45]. These factors suggest a potential ceiling effect, whereby participants’ high baseline activity may have limited the observable benefits of the intervention. Speculatively, the absence of improvements in gait performance due to the resistance training in both intervention groups might follow the law of diminishing returns, meaning that potential adaptations decrease with a higher fitness level [46].

Of note, the lower exercise intensity during resistance training was deliberately chosen to obtain reference data from normal participants for a companion investigation with patients with glaucoma. The present study was part of a larger randomized controlled trial investigating the effect of a MMI compared to a UMI in glaucoma patients, for which this approach was selected to keep intraocular pressure low [47]. Nevertheless, the analysis of the training progression using the external load data revealed an increase in external load over the three months period indicating neuromuscular adaptations. Although this analysis is only a crude measure of training-related strength increases, the potential neuromuscular adaptations have not led to improvements in gait performance.

Against our hypothesis, gait and cognitive performance were also not altered after the MMI. In this regard, Hamacher and colleagues [13] have shown that a six-month dancing program, which also requires the incorporation of cognitive demands into the motor task [48], significantly reduced the MTC_CoV_ compared to a combined exercises intervention in older adults (e.g., endurance training, strength training, flexibility training). This finding might be related to the fact that dancing requires the incorporation of cognitive demands into the motor task [48]. In contrast, in the Life Kinetik^®^ program, the cognitive tasks are used as distractors (additional) during the motor tasks and are thus not directly relevant for motor task completion [24]. Consequently exercises in which the cognitive task is a prerequisite for the motor task execution, might be better suited to improve gait performance compared to exercises using the cognitive task as a distractor during the motor task [24].

The present study has some limitations. First, single-task walking was always conducted before dual-task walking, which might have promoted sequential effects. However, the possible sequential effects were systematic as well as stable and might therefore have not affect group comparisons. Second, the sensitivity of the calculated gait parameters might be too low to detect differences in the dependent variables in our healthy elderly cohort. For instance, Kulmala et al. (2014) have shown that joint power of the lower extremities during walking are affected by age and shift from distal to proximal [49]. Therefore, it might be possible that other gait measures are more sensitive to the interventions used in healthy elderly people. However, it was shown that the calculated gait parameters are sensitive to differentiate between glaucoma patients and healthy controls (e.g., slower gait velocity [50] and higher MTC_CoV_ [30] in glaucoma patients). Third, the lack of improvements in gait performance might be due to a lack of isolated training of the triceps surae muscle, which is essential for plantar flexion and propulsive torque production during walking [51, 52]. This muscle group is also affected by the aging process resulting in shorter steps and a higher power production in the hip and knee joints [49]. Moreover, the triceps surae muscle is also important for remaining gait stability after stumbling [53]. Therefore, an increase in exercises dedicated to the plantar flexors is warranted, which might lead to positive changes in gait performance [51–53]. Fourth, initial cognitive status and risk of falls of the participants was not measured meaning that the theoretical association between these factors remains speculative in the study participants and is only based on the available literature [5, 24]. Thus, future studies should consider testing the cognitive status before and after the intervention. Fifth, the sensitivity of the cognitive tests might have been too low to detect improvements in executive functioning in our sample of active, healthy elderly. Therefore, future studies should record cortical brain activity (e.g., with functional near-infrared spectroscopy) during the gait and cognitive tests to monitor changes in brain activation due to the training interventions [54]. Sixth, due to the Covid-19 pandemic the recruitment of participants was problematic [55], making it not possible to involve more inactive and vulnerable elderly. Therefore, interpretation of the present results with regard to the latter population is complicated. Nevertheless, knowing the effects of the different interventions in active elderly is also an important finding to inform future studies with a higher sample size (e.g., for adjusting the exercise protocols in terms of intensity).

In conclusion, the present study showed that neither a 12-week MMI nor UMI seems to have an impact on specific gait parameters (i.e., stride length, gait velocity, MTC) or their respective CoVs in physically active healthy elderly participants.

## Supplementary Information

Supplementary Material 1.

Supplementary Material 2.

Supplementary Material 3.

## Data Availability

Data are available on reasonable request to the corresponding author.

## Abbreviations

ANCOVA: Analysis of covariance
CoV: Coefficient of variation
DT: Dual task
DTC: Dual-task costs
Hz: Herz
MMI: Multimodal Intervention
MTC: Minimum toe clearance
RPE: Received perception scale rating on a perceived exertion
ST: Single task
UMI: Unimodal intervention
95% CI: 95% confidence interval

## References

[1] Izquierdo M, et al. International exercise recommendations in older adults (ICFSR): expert consensus guidelines. J Nutr Health Aging. 2021;25:824–53. 10.1007/s12603-021-1665-8. PMC12369211 34409961

[2] Clark BC, Manini TM. What is dynapenia? Nutrition (Burbank, Los Angeles County, Calif.). 2012;28:495–503. 10.1016/j.nut.2011.12.002. PMC3571692 22469110

[3] Mau-Moeller A, Behrens M, Lindner T, Bader R, Bruhn S. Age-related changes in neuromuscular function of the quadriceps muscle in physically active adults. J Electromyogr Kinesiology: Official J Int Soc Electrophysiological Kinesiol. 2013;23:640–8. 10.1016/j.jelekin.2013.01.009. 23453325

[4] Cohen JA, Verghese J, Zwerling JL. Cognition and gait in older people. Maturitas. 2016;93:73–7. 10.1016/j.maturitas.2016.05.005. 27240713

[5] MacAulay RK, et al. Longitudinal assessment of neuropsychological and temporal/spatial gait characteristics of elderly fallers: taking it all in Stride. Front Aging Neurosci. 2015;7:34. 10.3389/fnagi.2015.00034. PMC4364254 25852548

[6] Fragala MS, et al. Resistance Training for Older Adults: Position Statement From the National Strength and Conditioning Association. J Strength Cond Res. 2019;33:2019–52. 10.1519/JSC.0000000000003230. 31343601

[7] Statistisches Bundesamt. Statistischer Bericht - Todesursachen in Deutschland – 2023. 2024.

[8] Hamacher D, Hamacher D, Schega L. Towards the importance of minimum toe clearance in level ground walking in a healthy elderly population. Gait Posture. 2014;40:727–9. 10.1016/j.gaitpost.2014.07.016. 25128155

[9] Hausdorff JM, Rios DA, Edelberg HK. Gait variability and fall risk in community-living older adults: a 1-year prospective study. Arch Phys Med Rehabil. 2001;82:1050–6. 10.1053/apmr.2001.24893. 11494184

[10] Barrett RS, Mills PM, Begg RK. A systematic review of the effect of ageing and falls history on minimum foot clearance characteristics during level walking. Gait Posture. 2010;32:429–35. 10.1016/j.gaitpost.2010.07.010 20692163

[11] Hamacher D, Hamacher D, Herold F, Schega L. Are there differences in the dual-task walking variability of minimum toe clearance in chronic low back pain patients and healthy controls? Gait Posture. 2016;49:97–101. 10.1016/j.gaitpost.2016.06.026. 27395449

[12] Hamacher D, Hamacher D, Schega L. A cognitive dual task affects gait variability in patients suffering from chronic low back pain. Exp Brain Res. 2014;232:3509–13. 10.1007/s00221-014-4039-1. 25059910

[13] Hamacher D, Hamacher D, Rehfeld K, Hökelmann A, Schega L. The effect of a Six-Month dancing program on Motor-Cognitive Dual-Task performance in older adults. J Aging Phys Act. 2015;23:647–52. 10.1123/japa.2014-0067. 25642826

[14] Hamacher D, Hamacher D, Herold F, Schega L. Effect of dual tasks on gait variability in walking to auditory cues in older and young individuals. Exp Brain Res. 2016;234:3555–63. 10.1007/s00221-016-4754-x. 27534860

[15] Hamacher D, et al. Motor-cognitive dual-tasking under hypoxia. Exp Brain Res. 2017;235:2997–3001. 10.1007/s00221-017-5036-y. 28721516

[16] Broscheid K-C, Behrens M, Dettmers C, Jöbges M, Schega L. Effects of a 6-Min treadmill walking test on Dual-Task gait performance and prefrontal hemodynamics in people with multiple sclerosis. Front Neurol. 2022;13:822952. 10.3389/fneur.2022.822952. PMC9022001 35463151

[17] Hamacher D, Hamacher D, Müller R, Schega L, Zech A. The effect of a cognitive dual task on the control of minimum toe clearance while walking. Motor Control. 2019;23:344–53. 10.1123/mc.2018-0006. 30599803

[18] Leone C, et al. Cognitive-motor dual-task interference: A systematic review of neural correlates. Neurosci Biobehav Rev. 2017;75:348–60. 10.1016/j.neubiorev.2017.01.010. 28104413

[19] Muir-Hunter SW, Wittwer JE. Dual-task testing to predict falls in community-dwelling older adults: a systematic review. Physiotherapy. 2016;102:29–40. 10.1016/j.physio.2015.04.011. 26390824

[20] Deshpande N, et al. Psychological, physical, and sensory correlates of fear of falling and consequent activity restriction in the elderly: the InCHIANTI study. Am J Phys Med Rehabil. 2008;87:354–62. 10.1097/PHM.0b013e31815e6e9b. PMC2495025 18174852

[21] Falck RS, Davis JC, Liu-Ambrose T. What is the association between sedentary behaviour and cognitive function? A systematic review. Br J Sports Med. 2017;51:800–11. 10.1136/bjsports-2015-095551. 27153869

[22] Keating CJ, et al. Influence of resistance training on gait & balance parameters in older adults: A systematic review. Int J Environ Res Public Health. 2021;18. 10.3390/ijerph18041759. PMC7918150 33670281

[23] Herold F, Törpel A, Schega L, Müller NG. Functional and/or structural brain changes in response to resistance exercises and resistance training lead to cognitive improvements - a systematic review. Eur Rev Aging Phys Activity: Official J Eur Group Res Elder Phys Activity. 2019;16. 10.1186/s11556-019-0217-2. PMC6617693 31333805

[24] Herold F, Hamacher D, Schega L, Müller NG. Thinking while moving or moving while Thinking - Concepts of Motor-Cognitive training for cognitive performance enhancement. Front Aging Neurosci. 2018;10:228. 10.3389/fnagi.2018.00228. PMC6089337 30127732

[25] Hötting K, Röder B. Beneficial effects of physical exercise on neuroplasticity and cognition. Neurosci Biobehav Rev. 2013;37:2243–57. 10.1016/j.neubiorev.2013.04.005. 23623982

[26] Tait JL, Duckham RL, Milte CM, Main LC, Daly RM. Influence of sequential vs. Simultaneous Dual-Task exercise training on cognitive function in older adults. Front Aging Neurosci. 2017;9:368. 10.3389/fnagi.2017.00368. PMC5681915 29163146

[27] Bliss ES, Wong RHX, Howe PRC, Mills DE. Benefits of exercise training on cerebrovascular and cognitive function in ageing. J Cereb Blood Flow Metab. 2021;41:447–70. 10.1177/0271678X20957807. PMC7907999 32954902

[28] Lauenroth A, Ioannidis AE, Teichmann B. Influence of combined physical and cognitive training on cognition: a systematic review. BMC Geriatr. 2016;16:141. 10.1186/s12877-016-0315-1. PMC4950255 27431673

[29] Law LLF, Barnett F, Yau MK, Gray MA. Effects of combined cognitive and exercise interventions on cognition in older adults with and without cognitive impairment: a systematic review. Ageing Res Rev. 2014;15:61–75. 10.1016/j.arr.2014.02.008. 24632497

[30] Freitag CW, et al. Single- and Dual-Task gait performance in patients with Open-Angle glaucoma: A Cross-sectional study. Translational Vis Sci Technol. 2023;12:31. 10.1167/tvst.12.11.31. PMC10691400 38015169

[31] Eldridge SM, et al. CONSORT 2010 statement: extension to randomised pilot and feasibility trials. BMJ. 2016;355:i5239. 10.1136/bmj.i5239. PMC5076380 27777223

[32] Demirakca T, Cardinale V, Dehn S, Ruf M, Ende G. The Exercising Brain: Changes in Functional Connectivity Induced by an Integrated Multimodal Cognitive and Whole-Body Coordination Training. Neural Plast. 2016;2016:8240894. 10.1155/2016/8240894. PMC4706972 26819776

[33] Frey I, Berg A, Grathwohl D, Keul J. Freiburger Fragebogen Zur körperlichen Aktivität–Entwicklung, Prüfung und Anwendung. Soz Praventivmed. 1999;44:55–64. 10.1007/BF01667127. 10407953

[34] Lutz H. Life Kinetik^®^. Gehirntraining durch Bewegung. 8th ed. München: BLV; 2021.

[35] Pageaux B. Perception of effort in exercise science: Definition, measurement and perspectives. Eur J Sport Sci. 2016;16:885–94. 10.1080/17461391.2016.1188992. 27240002

[36] Lara PM, et al. Influence of the body positions adopted for resistance training on intraocular pressure: a comparison between the supine and seated positions. Graefe’s Archive Clin Experimental Ophthalmol = Albrecht Von Graefes Archiv Fur Klinische Und Experimentelle Ophthalmologie. 2023;261:1971–8. 10.1007/s00417-023-06009-0. PMC10272256 36795163

[37] Najmanová E, Pluháček F, Haklová M. Intraocular pressure response affected by changing of sitting and supine positions. Acta Ophthalmol. 2020;98:e368–72. 10.1111/aos.14267. PMC7216979 31602816

[38] European Glaucoma Society Terminology and Guidelines for Glaucoma. 5th Edition. Br J Ophthalmol*.* 2021;105:1–169. 10.1136/bjophthalmol-2021-egsguidelines. 34675001

[39] Essentials of Strength Training and Conditioning. 4th Edition. Medicine & Science in Sports & Exercise. 2016;48:2073. 10.1249/MSS.0000000000001081.

[40] Vickers AJ. Parametric versus non-parametric statistics in the analysis of randomized trials with non-normally distributed data. BMC Med Res Methodol. 2005;5:35. 10.1186/1471-2288-5-35. PMC1310536 16269081

[41] Lakens D. Calculating and reporting effect sizes to facilitate cumulative science: a practical primer for t-tests and ANOVAs. Front Psychol. 2013;4:863. 10.3389/fpsyg.2013.00863. PMC3840331 24324449

[42] Wollesen B, et al. Effects of Dual-Task management and resistance training on gait performance in older individuals: A randomized controlled trial. Front Aging Neurosci. 2017;9:415. 10.3389/fnagi.2017.00415. PMC5733355 29326581

[43] Fiatarone Singh MA, et al. The study of mental and resistance training (SMART) study—resistance training and/or cognitive training in mild cognitive impairment: a randomized, double-blind, double-sham controlled trial. J Am Med Dir Assoc. 2014;15:873–80. 10.1016/j.jamda.2014.09.010. 25444575

[44] Castaño LAA, et al. Resistance training combined with cognitive training increases brain derived neurotrophic factor and improves cognitive function in healthy older adults. Front Psychol. 2022;13:870561. 10.3389/fpsyg.2022.870561. PMC9613948 36312128

[45] Oh-Park M, Holtzer R, Xue X, Verghese J. Conventional and robust quantitative gait norms in community-dwelling older adults. J Am Geriatr Soc. 2010;58:1512–8. 10.1111/j.1532-5415.2010.02962.x. PMC2955162 20646103

[46] McMaster DT, Gill N, Cronin J, McGuigan M. The development, retention and decay rates of strength and power in elite rugby union, rugby league and American football: a systematic review. Sports Med. 2013;43:367–84. 10.1007/s40279-013-0031-3. 23529287

[47] Ong SR, Crowston JG, Loprinzi PD, Ramulu PY. Physical activity, visual impairment, and eye disease. Eye. 2018;32:1296–303. 10.1038/s41433-018-0081-8. PMC6085324 29610523

[48] Hamacher D, Hamacher D, Rehfeld K, Schega L. Motor-cognitive dual-task training improves local dynamic stability of normal walking in older individuals. Clin Biomech (Bristol Avon). 2016;32:138–41. 10.1016/j.clinbiomech.2015.11.021 26682629

[49] Kulmala J-P, et al. Which muscles compromise human locomotor performance with age? J R Soc Interface. 2014;11:20140858. 10.1098/rsif.2014.0858. PMC4191118 25209406

[50] Lee H-S, et al. Gait characteristics during crossing over obstacle in patients with glaucoma using insole foot pressure. Medicine. 2021;100:e26938. 10.1097/MD.0000000000026938. PMC8360450 34397944

[51] Burke R, et al. Exercise selection differentially influences lower body regional muscle development. J SCI SPORT Exerc. 2024. 10.1007/s42978-024-00299-4.

[52] Kinoshita M, et al. Triceps Surae muscle hypertrophy is greater after standing versus seated calf-raise training. Front Physiol. 2023;14:1272106. 10.3389/fphys.2023.1272106. PMC10753835 38156065

[53] Pijnappels M, Bobbert MF, van Dieën JH. Control of support limb muscles in recovery after tripping in young and older subjects. Exp Brain Res. 2005;160:326–33. 10.1007/s00221-004-2014-y. 15322782

[54] Nguyen T, et al. Associations between gait performance and pain intensity, psychosocial factors, executive functions as well as prefrontal cortex activity in chronic low back pain patients: A cross-sectional fNIRS study. Front Med. 2023;10:1147907. 10.3389/fmed.2023.1147907. PMC10196398 37215712

[55] Xue JZ, et al. Clinical trial recovery from COVID-19 disruption. Nat Rev Drug Discov. 2020;19:662–3. 10.1038/d41573-020-00150-9. 32913212

