# Correction: Neither uni- nor multi-modal exercise interventions improved single- and dual-task gait performance in physically active healthy elderly – a pilot study

**DOI:** 10.1186/s12877-026-08339-0

**Published:** 2026-09-26

**Authors:** Constantin W. Freitag, Martin Behrens, Robert Bielitzki, Tom Behrendt, Khaldoon O. Al-Nosairy, Francie H. Stolle, Gokulraj T. Prabhakaran, Rosalie Beyer, Cynthia Moffack Djuloun, Hagen Thieme, Michael B. Hoffmann, Lutz Schega

**Affiliations:** 1 Department of Sport Science, Institute III, Otto von Guericke University Magdeburg, Zschokkestraße 32, Magdeburg, 39104 Germany; 2 University of Applied Sciences for Sport and Management Potsdam, Olympischer Weg 7, Potsdam, 14471 Germany; 3 Department of Orthopaedics, Rostock University Medical Center, Rostock, 18057 Germany; 4 Ophthalmic Department, Section for Clinical and Experimental Sensory Physiology, University Hospital Magdeburg, Leipziger Str. 44, Magdeburg, 39120 Germany; 5 Center for Behavioral Brain Research, Magdeburg, 39106 Germany

**Correction: BMC Geriatr 25, 840 (2025)**

**https://doi.org/10.1186/s12877-025-06537-w**

Following publication of the original article, the authors would like to correct the data on page 5 under the heading Intervention.

“7 repetitions” should be “20 repetitions”.

The original article [1] has been updated.
