# Supplementary material for: Neither uni- nor multi-modal exercise interventions improved single- and dual-task gait performance in physically active healthy elderly – a pilot study
Source: BMC Geriatr. 2025 Nov 4;25:840. doi: 10.1186/s12877-025-06537-w (PMC12584342; doi:10.1186/s12877-025-06537-w)
Supplement: Supplementary file 1 — Supplementary Material 1. [file 12877_2025_6537_MOESM1_ESM.docx]

| Outcome Measures | N (MMI-UMI) | p | $\eta_{p}^{2}$ | Achieved Power | Required Total Sample Size |
| --- | --- | --- | --- | --- | --- |
| Stride Length (m) | 11 - 13 | 0.613 | 0.032 | 0.399 | 102 |
| Stride Length_CoV_ (%) | 11 - 13 | 0.801 | 0.021 | 0.279 | 154 |
| Gait Velocity (m/s) | 11 - 13 | 0.675 | 0.025 | 0.323 | 130 |
| Gait Velocity_CoV_ (%) | 11 – 13 | 0.874 | 0.015 | 0.212 | 216 |
| MTC (cm) | 10 - 13 | 0.444 | 0.049 | 0.547 | 66 |
| MTC_CoV_ (%) | 10 - 13 | 0.098 | 0.104 | 0.876 | 32 |
| Cognitive Performance | 11 – 13 | 0.435 | 0.047 | 0.548 | 68 |

Table A. Post-hoc power and sample size calculation for the different gait parameters based on the TIME x INTERVENTION x CONDITION effects

Post-hoc power analysis(test family = F-test, statistical test = repeated measures ANOVA, type of power analysis = post hoc, α = 0.05, power (1 – β error probability) threshold = 0.95, sample size = 24, number of groups = 2, number of measurements = 2, correction among repeated measures = 0.5, ϵ = 1) and sample size calculation (test family = F-test, statistical test = repeated measures ANOVA, type of power analysis = post hoc, α = 0.05, power (1 – β error probability) threshold = 0.95, number of groups = 2, number of measurements = 2, correction among repeated measures = 0.5, ϵ = 1) based on the found TIME x INTERVENTION x CONDITION effect sizes for the different outcomes.

Table B. Post-hoc power and sample size calculation for the different dual-task costs (DTC) based on the TIME x INTERVENTION x CONDITION effects

| Outcome Measures | N (MMI-UMI) | p | $\eta_{p}^{2}$ | Achieved Power | Required Total Sample Size |
| --- | --- | --- | --- | --- | --- |
| DTC Stride Length (m) | 11 – 12 | 0.52 | 0.04 | 0.463 | 80 |
| DTC Stride Length_CoV_ (%) | 11 – 12 | 0.724 | 0.022 | 0.279 | 148 |
| DTC Gait Velocity (m/s) | 11 – 12 | 0.544 | 0.036 | 0.424 | 90 |
| DTC Gait Velocity_CoV_ (%) | 11 – 12 | 0.577 | 0.036 | 0.424 | 90 |
| DTC MTC (cm) | 10 - 12 | 0.162 | 0.093 | 0.815 | 34 |
| DTC MTC_CoV_ (%) | 10 - 12 | 0.073 | 0.128 | 0.927 | 26 |
| DTC Cognitive Performance | 11 – 12 | 0.680 | 0.014 | 0.194 | 232 |

Post-hoc power analysis (test family = F-test, statistical test = repeated measures ANOVA, type of power analysis = post hoc, α = 0.05, power (1 – β error probability) threshold = 0.95, sample size = 23, number of groups = 2, number of measurements = 2, correction among repeated measures = 0.5, ϵ = 1) and sample size calculation (test family = F-test, statistical test = repeated measures ANOVA, type of power analysis = post hoc, α = 0.05, power (1 – β error probability) threshold = 0.95, number of groups = 2, number of measurements = 2, correction among repeated measures = 0.5, ϵ = 1) based on the found TIME x INTERVENTION x CONDITION effect sizes for the different outcomes.

Table C. Means ± standard deviations of the pre and post gait assessment as well as the outcomes of the ANCOVA

| Time | Condition/  Intervention | Single -Task Walking | Letter Fluency Task  (easy) | Letter Fluency Task  (severe) | Reaction  Time  Task  (easy) | Reaction  Time Task  (severe) | N-Back  Task  (easy) | N-Back  Task  (severe) | Repeated Measures ANCOVA | | |
| --- | --- | --- | --- | --- | --- | --- | --- | --- | --- | --- | --- |
|  |  |  |  |  |  |  |  |  | ***Time*** | ***Time x Intervention*** | ***Time x Intervention x Condition*** |
|  | **MTC (cm)** | | | | | | | | | | |
| PRE | MMI  N = 10 | 2.393  ± 0.508 | 2.013  ± 0.472 | 1.893  ± 0.464 | 2.208  ± 0.509 | 2.199  ± 0.502 | 2.162  ± 0.516 | 2.011  ± 0.547 | F_1,18_ = 0.000,  p = 0.996,  $\eta_{p}^{2}$ = 0.000 | F_1,18_ = 1.689,  p = 0.210,  $\eta_{p}^{2}$ = 0.086 | F_3.968,66.558_ = 0.935,  p = 0.444,  $\eta_{p}^{2}$ = 0.049 |
|  | UMI  N = 13 | 2.571  ± 0.536 | 2.081  ± 0.440 | 2.022  ± 0.445 | 2.320  ± 0.455 | 2.162  ± 0.499 | 2.296  ±0.506 | 2.178  ± 0.534 |  |  |  |
| POST | MMI  N = 10 | 2.242  ± 0.450 | 1.897  ± 0.415 | 1.804  ± 0.394 | 2.142  ± 0.373 | 2.051  ± 0.406 | 2.072  ± 0.405 | 1.928  ± 0.404 |  |  |  |
|  | UMI  N = 13 | 2.384  ± 0.523 | 1.955  ± 0.501 | 1.907  ± 0.485 | 2.252  ± 0.460 | 2.150  ± 0.464 | 2.173  ± 0.546 | 2.146  ± 0.522 |  |  |  |
|  | **MTC_CoV_ (%)** | | | | | | | | | | |
| PRE | MMI  N = 10 | 28.300  ± 5.674 | 31.052  ± 9.123 | 31.014  ± 7.929 | 27.041  ± 9.123 | 25.658  ± 3.517 | 30.499  ± 9.277 | 29.956  ± 7.754 | F_1,18_ = 0.031,  p = 0.862,  $\eta_{p}^{2}$ = 0.002 | F_1,18_ = 0.045,  p = 0.834,  $\eta_{p}^{2}$ = 0.002 | F_3.696,66.529_ = 2.080,  p = 0.098,  $\eta_{p}^{2}$ = 0.104 |
|  | UMI  N = 13 | 31.563  ± 7.822 | 30.382  ± 8.083 | 30.410  ± 9.934 | 29.775  ± 25.657 | 32.003  ± 7.363 | 30.914  ± 8.000 | 33.165  ± 9.670 |  |  |  |
| POST | MMI  N = 10 | 31.714  ± 9.216 | 33.301  ± 10.219 | 31.012±  8.607 | 30.137  ± 7.128 | 30.113  ± 8.298 | 30.236  ± 8.575 | 30.884  ± 8.400 |  |  |  |
|  | UMI  N = 13 | 34.426  ± 9.008 | 34.164  ± 8.405 | 32.947  ± 6.575 | 31.411  ± 7.469 | 32.341  ± 7.747 | 34.704  ± 9.159 | 34.074  ± 9.093 |  |  |  |
|  | **Stride Length (cm)** | | | | | | | | | | |
| PRE | MMI  N = 11 | 1.289  ± 0.072 | 1.202  ± 0.120 | 1.189  ± 0.122 | 1.237  ± 0.112 | 1.253  ± 0.108 | 1.251  ± 0.104 | 1.223  ± 0.103 | F_1,19_ = 1.294,  p = 0.269,  $\eta_{p}^{2}$ = 0.064 | F_1,19_ = 0.341,  p = 0.566,  $\eta_{p}^{2}$ = 0.018 | F_3.491,66.334_ = 0.001,  p = 0.613,  $\eta_{p}^{2}$ = 0.033 |
|  | UMI  N = 13 | 1.356  ± 0.128 | 1.240  ± 0.114 | 1.236  ± 0.112 | 1.296  ± 0.172 | 1.290  ± 0.117 | 1.293  ± 0.117 | 1.271  ± 0.123 |  |  |  |
| POST | MMI  N =10 | 1.275  ± 0.102 | 1.189  ± 0.112 | 1.190  ± 0.112 | 1.238  ± 0.115 | 1.244  ± 0.118 | 1.236  ± 0.108 | 1.213  ± 0.104 |  |  |  |
|  | UMI  N = 13 | 1.328  ± 0.118 | 1.236  ± 0.106 | 1.236  ± 0.108 | 1.277  ± 0.109 | 1.273  ± 0.109 | 1.283  ± 0.131 | 1.272  ± 0.124 |  |  |  |
|  | **Stride Length_CoV_ (%)** | | | | | | | | | | |
| PRE | MMI  N = 11 | 13.668  ± 2.443 | 13.297  ± 2.215 | 12.460  ± 2.723 | 12.914  ± 2.411 | 12.544  ± 2.625 | 12.469  ± 2.600 | 12.450  ± 3.031 | F_1,19_ = 0.001,  p = 0.982,  $\eta_{p}^{2}$ = 0.000 | F_1,19_ = 0.176,  p = 0.680,  $\eta_{p}^{2}$ = 0.009 | F_3.908,74.257_ = 0.404,  p = 0.801,  $\eta_{p}^{2}$ = 0.021 |
|  | UMI  N = 13 | 15.451  ± 3.012 | 13.534  ± 2.704 | 13.591  ± 3.264 | 12.958  ± 2.568 | 14.419  ± 2.667 | 13.528  ± 2.782 | 13.830  ± 2.847 |  |  |  |
| POST | MMI  N = 11 | 13.374  ± 2.296 | 12.823  ± 2.968 | 11.624  ± 2.851 | 12.622  ± 2.223 | 12.168  ± 2.125 | 12.403  ± 1.899 | 12.375  ± 2.348 |  |  |  |
|  | UMI  N = 13 | 15.093  ± 2.537 | 13.508  ± 2.841 | 13.458  ± 2.695 | 13.306  ± 2.413 | 13.582  ± 2.216 | 14.490  ± 2.380 | 14.422  ± 2.046 |  |  |  |
|  | **Gait Velocity (m/s)** | | | | | | | | | | |
| PRE | MMI  N = 11 | 1.246  ± 0.097 | 1.075  ± 0.209 | 1.037  ± 0.199 | 1.177  ± 0.172 | 1.195  ± 0.173 | 1.179  ± 0.155 | 1.123  ± 0.161 | F_1,19_ = 2.560,  p = 0.126,  $\eta_{p}^{2}$ = 0.119 | F_1,19_ = 1.360,  p = 0.258,  $\eta_{p}^{2}$ = 0.067 | F_2.708,51.450_ = 0.485,  p = 0.675,  $\eta_{p}^{2}$ = 0.025 |
|  | UMI  N = 13 | 1.274  ± 0.130 | 1.080  ± 0.102 | 1.077  ± 0.107 | 1.210  ± 0.133 | 1.186  ± 0.123 | 1.195  ± 0.136 | 1.159  ± 0.138 |  |  |  |
| POST | MMI  N = 11 | 1.237  ± 0.125 | 1.062  ± 0.148 | 1.044  ± 0.173 | 1.179  ± 0.164 | 1.187  ± 0.177 | 1.168  ± 0.153 | 1.122  ± 0.138 |  |  |  |
|  | UMI  N = 13 | 1.258  ± 0.140 | 1.096  ± 0.103 | 1.084  ± 0.109 | 1.176  ± 0.136 | 1.177  ± 0.149 | 1.200  ± 0.172 | 1.175  ± 0.157 |  |  |  |
|  | **Gait Velocity_CoV_ (%)** | | | | | | | | | | |
| PRE | MMI  N = 11 | 16.043  ± 2.917 | 15.743  ± 2.283 | 14.897  ± 2.648 | 15.240  ± 3.101 | 15.076  ± 3.508 | 14.560  ± 2.945 | 14.653  ± 3.207 | F_1,19_ = 0.011  p = 0.917,  $\eta_{p}^{2}$ = 0.001 | F_1,19_ = 0.340,  p = 0.566,  $\eta_{p}^{2}$ = 0.018 | F_3.803,72.248_ = 0.344,  p = 0.838,  $\eta_{p}^{2}$ = 0.018 |
|  | UMI  N = 13 | 17.283  ± 3.265 | 15.454  ± 3.137 | 15.300  ± 3.625 | 14.504  ± 2.842 | 15.883  ± 3.212 | 15.009  ± 3.322 | 15.316  ± 3.202 |  |  |  |
| POST | MMI  N = 11 | 15.540  ± 2.744 | 15.460  ± 3.127 | 14.047  ± 3.136 | 14.814  ± 2.849 | 14.354  ± 2.988 | 14.313  ± 2.521 | 14.503  ± 2.764 |  |  |  |
|  | UMI  N = 13 | 17.060  ± 3.120 | 15.497  ± 3.188 | 15.440  ± 3.223 | 14.741  ± 2.870 | 15.369  ± 2.744 | 15.992  ± 2.718 | 16.116  ± 2.467 |  |  |  |

Table D. Means ± standard deviations for the cognitive and motor dual task costs (DTC) of the pre and post measurement as well as the outcomes of the ANCOVA

| Time | Condition/  Group | Letter Fluency Task  (easy) | Letter Fluency Task  (severe) | Reaction  Time  Task  (easy) | Reaction  Time Task  (severe) | N-Back  Task  (easy) | N-Back  Task  (severe) | DTC Repeated Measures ANCOVA | | |
| --- | --- | --- | --- | --- | --- | --- | --- | --- | --- | --- |
|  |  |  |  |  |  |  |  | ***Time*** | ***Time x Intervention*** | ***Time x Intervention x Condition*** |
|  | **DTC MTC (%)** | | | | | | | | | |
| PRE | MMI  N = 10 | 15.925  ± 8.615 | 20.772  ±10.747 | 4.572  ± 5.401 | 4.612  ±10.721 | 8.787  ±10.580 | 15.455  ± 13.691 | F_1,17_ = 4.070,  p = 0.060,  $\eta_{p}^{2}$ = 0.193 | F_1,17_ = 0.369,  p = 0.552,  $\eta_{p}^{2}$ = 0.021 | F_3.250,55.245_ = 1.682,  p = 0.178,  $\eta_{p}^{2}$ = 0.090 |
|  | UMI  N = 12 | 18.689  ± 9.386 | 20.888  ± 8.115 | 10.047  ± 7.575 | 16.257  ±10.626 | 6.180  ±10.650 | 10.911  ± 13.058 |  |  |  |
| POST | MMI  N = 10 | 16.643  ±12.405 | 20.031  ±17.747 | 4.123  ± 4.532 | 8.438  ± 6.316 | 8.469  ± 6.382 | 14.700  ± 10.822 |  |  |  |
|  | UMI  N = 12 | 17.152  ± 10.186 | 19.134  ± 6.511 | 5.977  ± 9.214 | 10.461  ± 6.560 | 11.613  ± 6.576 | 12.301  ± 6.594 |  |  |  |
|  | **DTC MTC_CoV_ (%)** | | | | | | | | | |
| PRE | MMI  N = 10 | 0.582  ± 17.511 | 1.920  ± 14.343 | -6.660  ± 13.257 | -12.308  ± 18.257 | 2.566  ± 13.258 | 1.425  ± 14.396 | F_1,17_ = 3.203,  p = 0.091,  $\eta_{p}^{2}$ = 0.159 | F_1,17_ = 0.359,  p = 0.557,  $\eta_{p}^{2}$ = 0.021 | F_2.864,48.687_ = 2.418  p = 0.080 $\eta_{p}^{2}$ = 0.125 |
|  | UMI  N = 12 | -2.633  ± 20.417 | -4.379  ± 15.037 | -2.065  ±12.706 | 6.814  ± 9.347 | -7.288  ± 16.951 | 0.110  ± 13.761 |  |  |  |
| POST | MMI  N = 10 | 2.406  ± 15.809 | -5.590  ± 33.308 | 1.395  ± 6.932 | 0.631  ± 5.844 | -6.130  ± 13.228 | -3.571  ± 10.849 |  |  |  |
|  | UMI  N = 12 | 0.163  ± 13.970 | -2.470  ± 15.483 | -4.867  ± 19.685 | -1.633  ± 12.531 | -0.798  ± 11.725 | -2.837  ± 14.329 |  |  |  |
| **DTC Stride Length (%)** | | | | | | | | | | |
| PRE | MMI  N = 11 | 6.513  ± 5.912 | 7.536  ± 5.887 | 2.548  ± 4.501 | 1.144  ± 6.319 | 2.498  ± 5.220 | 4.637  ± 5.586 | F_1,18_ = 0.009,  p = 0.924,  $\eta_{p}^{2}$ = 0.000 | F_1,18_ = 0.486,  p = 0.495,  $\eta_{p}^{2}$ = 0.026 | F_2.724,49.031_ = 0.742,  p = 0.520,  $\eta_{p}^{2}$ = 0.040 |
|  | UMI  N = 12 | 8.149  ± 4.558 | 8.558  ± 4.137 | 4.279  ± 3.593 | 4.737  ± 3.529 | 3.864  ± 1.939 | 5.380  ± 1.344 |  |  |  |
| POST | MMI  N = 11 | 7.090  ± 3.074 | 7.092  ± 5.362 | 2.230  ± 2.560 | 1.776  ± 3.336 | 2.535  ± 3.015 | 4.252  ± 4.071 |  |  |  |
|  | UMI  N = 12 | 7.175  ± 3.459 | 7.543  ± 3.600 | 3.477  ± 3.637 | 3.850  ± 2.239 | 4.035  ± 2.018 | 4.797  ± 1.743 |  |  |  |
| **DTC Stride Length_CoV_ (%)** | | | | | | | | | | |
| PRE | MMI  N = 11 | -5.908  ± 11.387 | -13.910  ± 12.454 | -13.395  ± 18.980 | -17.813  ± 22.586 | -9.605  ± 16.998 | -11.478  ± 24.058 | F_1,18_ = 0.024,  p = 0.879,  $\eta_{p}^{2}$ = 0.001 | F_1,18_ = 0.011,  p = 0.919,  $\eta_{p}^{2}$ = 0.001 | F_2.675,48.149_ = 0.409,  p = 0.724,  $\eta_{p}^{2}$ = 0.022 |
|  | UMI  N = 12 | -10.071  ± 13.952 | -10.664  ± 15.259 | -14.820  ± 12.740 | -4.332  ± 21.552 | -12.711  ± 9.600 | -10.640  ± 8.227 |  |  |  |
| POST | MMI  N = 11 | -4.796  ± 11.054 | -16.729  ± 20.075 | -6.180  ± 13.55 | -10.069  ± 13.060 | -10.146  ± 12.610 | -11.077  ± 12.887 |  |  |  |
|  | UMI  N = 12 | -6.541  ± 14.569 | -6.458  ± 14.628 | -13.859  ± 7.811 | -10.971  ± 8.502 | -8.256  ± 7.445 | -7.549  ± 8.200 |  |  |  |
| **DTC Gait Velocity (%)** | | | | | | | | | | |
| PRE | MMI  N = 11 | 13.834  ± 13.162 | 16.702  ± 13.346 | 3.661  ± 9.230 | 1.999  ± 11.052 | 4.310  ± 11.244 | 8.748  ± 12.638 | F_1,18_ = 0.100,  p = 0.755,  $\eta_{p}^{2}$ = 0.006 | F_1,18_ = 0.000,  p = 0.999,  $\eta_{p}^{2}$ = 0.006 | F_26.515,45.277_ = 0.679,  p = 0.544,  $\eta_{p}^{2}$ = 0.036 |
|  | UMI  N = 12 | 14.302  ± 8.447 | 14.715  ± 7.528 | 5.123  ± 5.394 | 6.965  ± 8.116 | 4.225  ± 2.490 | 6.783  ± 3.699 |  |  |  |
| POST | MMI  N = 11 | 14.411  ± 8.589 | 15.885  ± 11.079 | 3.210  ± 5.634 | 2.675  ± 7.260 | 4.330  ± 7.421 | 7.918  ± 8.644 |  |  |  |
|  | UMI  N = 12 | 12.702  ± 8.043 | 13.525  ± 8.289 | 5.512  ± 6.453 | 5.584  ± 4.818 | 5.123  ± 2.856 | 6.564  ± 4.126 |  |  |  |
| **DTC Gait Velocity_CoV_ (%)** | | | | | | | | | | |
| PRE | MMI  N = 11 | -3.877  ± 11.035 | -10.008  ± 9.063 | -10.636  ± 11.140 | -13.014  ± 15.192 | -10.037  ± 14.090 | -9.986  ± 16.756 | F_1,18_ = 0.041,  p = 0.842,  $\eta_{p}^{2}$ = 0.002 | F_1,18_ = 0.024,  p = 0.880,  $\eta_{p}^{2}$ = 0.001 | F_3.207,54.719_ = 0.681,  p = 0.577,  $\eta_{p}^{2}$ = 0.036 |
|  | UMI  N = 12 | -9.996  ± 12.002 | -11.997  ± 12.757 | -13.991  ± 11.515 | -5.315  ± 19.736 | -14.463  ± 12.686 | -12.266  ± 12.679 |  |  |  |
| POST | MMI  N = 11 | -0.442  ± 8.117 | -11.323  ± 15.310 | -6.875  ± 12.870 | -10.753  ± 13.805 | -9.726  ± 9.734 | -8.638  ± 9.516 |  |  |  |
|  | UMI  N = 12 | -4.831  ± 9.960 | -5.039  ± 11.012 | -16.615  ± 11.235 | -10.678  ± 9.084 | -10.805  ± 7.717 | -8.835  ± 9.923 |  |  |  |
| **DTC Cognitive Performance (%)** | | | | | | | | | | |
| PRE | MMI  N = 11 | 3.087  ± 20.880 | -5.866  ± 20.995 | 0.764  ± 9.634 | 4.657  ± 7.221 | -0.101  ± 4.230 | -33.857  ± 121.999 | F_1,19_ = 0.192,  p = 0.667,  $\eta_{p}^{2}$ = 0.010 | F_1,19_ = 0.418,  p = 0.525,  $\eta_{p}^{2}$ = 0.022 | F_1.314,66.430_ = 0.284,  p = 0.661,  $\eta_{p}^{2}$ = 0.015 |
|  | UMI  N = 13 | 3.521  ± 21.068 | -0.659  ± 21.909 | 6.378  ± 10.746 | 4.856  ± 8.269 | 3.661  ± 9.638 | -10.383  ± 28.600 |  |  |  |
| POST | MMI  N = 11 | 5.253  ± 15.332 | 3.495  ± 25.573 | 6.240  ± 5.945 | 5.559  ± 5.872 | -0.650  ± 1.448 | -1.411  ± 15.651 |  |  |  |
|  | UMI  N = 13 | 1.688  ± 12.868 | 5.764  ± 19.517 | 5.266  ± 7.317 | 9.219  ± 6.691 | -4.672  ± 9.420 | -8.143  ± 22.946 |  |  |  |

Table E. Means ± standard deviations for the cognitive performance measures performed during the pre and post measurements as well as the outcomes of the ANCOVA

| Time | Condition/ Intervention | Single-Task | | | | | | | Dual-Task | | | | | |
| --- | --- | --- | --- | --- | --- | --- | --- | --- | --- | --- | --- | --- | --- | --- |
|  |  | Letter Fluency Task (n)  (easy) | Letter Fluency Task (n)  (severe) | Reaction  Time  Task (s)  (easy) | | Reaction Time  Task (s)  (severe) | N-Back  Task  (n)  (easy) | N-Back  Task  (n)  (severe) | Letter Fluency Task  (n)  (easy) | Letter Fluency Task  (n)  (severe) | Reaction  Time  Task  (s)  (easy) | Reaction  Time  Task  (s)  (severe) | N-Back  Task  (n)  (easy) | N-Back  Task  (n)  (severe) |
| PRE | MMI  N = 11 | 37.000  ± 7.100 | 30.636  ± 8.201 | 1.004  ± 0.142 | | 0.907  ± 0.097 | 29.636  ± 0.924 | 22.545  ± 7.005 | 35.364  ± 8.401 | 31.545  ± 7.394 | 1.012  ± 0.114 | 0.955  ± 0.106 | 29.636  ± 0.674 | 23.363  ± 4.202 |
|  | UMI  N = 13 | 35.077  ± 13.524 | 27.308  ± 11.586 | 0.915  ± 0.160 | | 0.875  ± 0.142 | 29.231  ± 1.481 | 21.385  ± 5.409 | 32.692  ± 11.280 | 27.000  ± 11.136 | 0.982  ± 0.158 | 0.919  ± 0.128 | 28.077  ± 2.253 | 21.154  ± 5.669 |
| POST | MMI  N = 11 | 41.727  ± 7.030 | 35.182  ± 9.250 | 0.885  ± 0.205 | | 0.831  ± 0.201 | 29.636  ± 0.924 | 24.909  ± 3.936 | 39.545  ± 9.125 | 32.545  ± 6.424 | 0.946  ± 0.211 | 0.877  ± 0.194 | 29.818  ± 0.603 | 24.818  ± 2.786 |
|  | UMI  N = 13 | 38.385  ± 11.822 | 31.615  ± 8.520 | 0.928  ± 0.221 | | 0.856  ± 0.206 | 28.000  ± 3.240 | 22.923  ± 5.041 | 37.385  ± 10.813 | 29.462  ± 5.571 | 0.981  ± 0.221 | 0.945  ± 0.218 | 29.154  ± 2.764 | 24.154  ± 4.432 |
| Repeated Measures ANCOVA | | | | | | | | | | | | | | |
| ***Time*** | | | | | ***Time x Intervention*** | | | | | | ***Time x Intervention x Condition*** | | | |
| F_1,19_ = 1.071,  p = 0.313, $\eta_{p}^{2}$ = 0.053 | | | | | F_1,19_ = 1.795  p = 0.196, $\eta_{p}^{2}$ = 0.086 | | | | | | F_4.351,82.671_ = 0.894  p = 0.478, $\eta_{p}^{2}$ = 0.005 | | | |
