# Supplementary material for: Neither uni- nor multi-modal exercise interventions improved single- and dual-task gait performance in physically active healthy elderly – a pilot study
Source: BMC Geriatr. 2025 Nov 4;25:840. doi: 10.1186/s12877-025-06537-w (PMC12584342; doi:10.1186/s12877-025-06537-w)
Supplement: Supplementary file 2 — Supplementary Material 2. [file 12877_2025_6537_MOESM2_ESM.docx]

| Outcome Measures | N (UMI-MMI) | p | $\eta_{p}^{2}$ | Required Total Sample Size |
| --- | --- | --- | --- | --- |
| Stride Length (m) | 12 – 12 | 0.112 | 0.085 | 38 |
| Stride Length_CoV_ (%) | 12 – 12 | 0.435 | 0.044 | 74 |
| Gait Velocity (m/s) | 12 – 12 | 0.134 | 0.084 | 38 |
| Gait Velocity_CoV_ (%) | 12 – 12 | 0.589 | 0.033 | 98 |
| MTC (cm) | 12 – 11 | 0.431 | 0.046 | 70 |
| MTC_CoV_ (%) | 12 – 11 | 0.515 | 0.039 | 84 |
| Cognitive Performance | 11 – 12 | 0.249 | 0.012 | 270 |

Table A. Sample size calculation for the different gait parameters based on the TIME x INTERVENTION x CONDITION effects

Sample size calculation (test family = F-test, statistical test = repeated measures ANOVA, type of power analysis = post hoc, α = 0.05, power (1 – β error probability) threshold = 0.95, number of groups = 2, number of measurements = 2, correction among repeated measures = 0.5, ϵ = 1) based on the found TIME x INTERVENTION x CONDITION effect sizes for the different outcomes.

Table B. Sample size calculation for the different dual-task costs (DTC) based on the TIME x INTERVENTION x CONDITION effects

| Outcome Measures | N (UMI-MMI) | p | $\eta_{p}^{2}$ | Required Total Sample Size |
| --- | --- | --- | --- | --- |
| DTC Stride Length (m) | 11 – 12 | 0.199 | 0.074 | 44 |
| DTC Stride Length_CoV_ (%) | 11 – 12 | 0.086 | 0.103 | 32 |
| DTC Gait Velocity (m/s) | 11 – 12 | 0.109 | 0.096 | 34 |
| DTC Gait Velocity_CoV_ (%) | 11 – 12 | 0.066 | 0.108 | 30 |
| DTC MTC (cm) | 11 - 11 | 0.201 | 0.076 | 42 |
| DTC MTC_CoV_ (%) | 11 - 11 | 0.637 | 0.029 | 54 |
| DTC Cognitive Performance | 11 – 12 | 0.415 | 0.045 | 72 |

Sample size calculation (test family = F-test, statistical test = repeated measures ANOVA, type of power analysis = post hoc, α = 0.05, power (1 – β error probability) threshold = 0.95, number of groups = 2, number of measurements = 2, correction among repeated measures = 0.5, ϵ = 1) based on the found TIME x INTERVENTION x CONDITION effect sizes for the different outcomes.

Table C. Means ± standard deviations of the pre and post gait assessment as well as the outcomes of the ANCOVA

| Time | Condition/  Intervention | Single -Task Walking | Letter Fluency Task  (easy) | Letter Fluency Task  (severe) | Reaction  Time  Task  (easy) | Reaction  Time Task  (severe) | N-Back  Task  (easy) | N-Back  Task  (severe) | Repeated Measures ANCOVA | | |
| --- | --- | --- | --- | --- | --- | --- | --- | --- | --- | --- | --- |
|  |  |  |  |  |  |  |  |  | ***Time*** | ***Time x Intervention*** | ***Time x Intervention x Condition*** |
|  | **MTC (cm)** | | | | | | | | | | |
| PRE | UMI  N = 12 | 2.435  ± 0.506 | 1.946  ± 0.362 | 1.864  ± 0.358 | 2.210  ± 0.487 | 2.045  ± 0.490 | 2.182  ± 0.544 | 2.021  ± 0.537 | F_1,20_ = 4.254,  p = 0.052,  $\eta_{p}^{2}$ = 0.175 | F_1,20_ = 0.472,  p = 0.500,  $\eta_{p}^{2}$ = 0.023 | F_3.838,76.759_ = 0.963,  p = 0.431,  $\eta_{p}^{2}$ = 0.046 |
|  | MMI  N = 11 | 2.558  ± 0.552 | 2.167  ± 0.514 | 2.077  ± 0.523 | 2.338  ± 0.467 | 2.323  ± 0.467 | 2.300  ± 0.472 | 2.196  ± 0.540 |  |  |  |
| POST | UMI  N = 12 | 2.292  ± 0.467 | 1.811  ± 0.387 | 1.758  ± 0.383 | 2.161  ± 0.419 | 2.064  ± 0.435 | 2.105  ± 0.505 | 2.046  ± 0.524 |  |  |  |
|  | MMI  N = 11 | 2.355  ± 0.529 | 2.060  ± 0.508 | 1.976  ± 0.489 | 2.251  ± 0.434 | 2.154  ± 0.448 | 2.156  ± 0.479 | 2.057  ± 0.446 |  |  |  |
|  | **MTC_CoV_ (%)** | | | | | | | | | | |
| PRE | UMI  N = 12 | 31.620  ± 7.241 | 30.281  ± 7.911 | 30.421  ± 9.335 | 30.045  ± 7.517 | 31.568  ± 7.728 | 31.327  ± 8.818 | 32.441  ± 9.947 | F_1,20_ = 1.979,  p = 0.175,  $\eta_{p}^{2}$ = 0.090 | F_1,20_ = 0.147,  p = 0.706,  $\eta_{p}^{2}$ = 0.007 | F_3.743,74.863_ = 0.812,  p = 0.515,  $\eta_{p}^{2}$ = 0.039 |
|  | MMI  N = 11 | 28.535  ± 6.728 | 31.102  ± 9.184 | 30.947  ± 8.899 | 26.994  ± 4.975 | 26.709  ± 4.440 | 30.086  ± 8.243 | 31.037  ± 7.890 |  |  |  |
| POST | UMI  N = 12 | 33.839  ± 8.068 | 34.757  ± 8.917 | 32.846  ± 7.097 | 32.442  ± 7.364 | 33.319  ± 8.090 | 34.176  ± 8.600 | 33.194  ± 8.560 |  |  |  |
|  | MMI  N = 11 | 32.601  ± 10.271 | 32.733  ± 9.453 | 31.298  ± 7.994 | 29.128  ± 6.902 | 29.249  ± 7.436 | 31.218  ± 9.579 | 32.134  ± 9.341 |  |  |  |
|  | **Stride Length (cm)** | | | | | | | | | | |
| PRE | UMI  N = 12 | 1.333  ± 0.132 | 1.227  ± 0.107 | 1.216  ± 0.108 | 1.284  ± 0.133 | 1.277  ± 0.121 | 1.271  ± 0.127 | 1.247  ± 0.136 | F_1,21_ = 0.109,  p = 0.745,  $\eta_{p}^{2}$ = 0.005 | F_1,21_ = 0.249,  p = 0.623,  $\eta_{p}^{2}$ = 0.012 | F_3.848,80.800_ = 1.957,  p = 0.112,  $\eta_{p}^{2}$ = 0.085 |
|  | MMI  N = 12 | 1.318  ± 0.085 | 1.219  ± 0.128 | 1.213  ± 0.129 | 1.254  ± 0.113 | 1.269  ± 0.107 | 1.276  ± 0.098 | 1.251  ± 0.094 |  |  |  |
| POST | UMI  N =12 | 1.314  ± 0.120 | 1.212  ± 0.107 | 1.206  ± 0.110 | 1.269  ± 0.107 | 1.269  ± 0.106 | 1.277  ± 0.119 | 1.260  ± 0.126 |  |  |  |
|  | MMI  N = 12 | 1.294  ± 0.107 | 1.217  ± 0.117 | 1.218  ± 0.133 | 1.249  ± 0.118 | 1.250  ± 0.121 | 1.245  0.126 | 1.230  ± 0.110 |  |  |  |
|  | **Stride Length_CoV_ (%)** | | | | | | | | | | |
| PRE | UMI  N = 12 | 15.119  ± 3.371 | 13.251  ± 3.091 | 13.315  ± 3.902 | 13.163  ± 2.898 | 14.209  ± 3.005 | 13.578  ± 3.180 | 13.753  ± 3.477 | F_1,21_ = 0.003,  p = 0.957,  $\eta_{p}^{2}$ = 0.000 | F_1,21_ = 0.298,  p = 0.591,  $\eta_{p}^{2}$ = 0.014 | F_3.922,82.365_ = 0.956,  p = 0.435,  $\eta_{p}^{2}$ = 0.044 |
|  | MMI  N = 12 | 14.149  ± 2.274 | 13.600  ± 1.688 | 12.832  ± 1.924 | 12.713  ± 1.994 | 12.911  ± 2.445 | 12.508  ± 2.118 | 12.641  ± 2.338 |  |  |  |
| POST | UMI  N = 12 | 14.763  ± 2.942 | 13.706  ± 3.432 | 13.131  ± 3.199 | 13.227  ± 2.862 | 13.303  ± 2.598 | 14.477  ± 2.664 | 14.088  ± 2.686 |  |  |  |
|  | MMI  N = 12 | 13.848  ± 2.074 | 12.682  ± 2.172 | 12.104  ± 2.515 | 12.758  ± 1.669 | 12.565  ± 1.871 | 12.590  ± 1.667 | 12.880  ± 1.958 |  |  |  |
|  | **Gait Velocity (m/s)** | | | | | | | | | | |
| PRE | UMI  N = 12 | 1.333  ± 0.132 | 1.227  ± 0.107 | 1.216  ± 0.108 | 1.284  ± 0.133 | 1.277  ± 0.121 | 1.271  ± 0.127 | 1.247  ± 0.136 | F_1,21_ = 0.109,  p = 0.745,  $\eta_{p}^{2}$ = 0.005 | F_1,21_ = 249,  p = 0.623,  $\eta_{p}^{2}$ = 0.012 | F_3.848,80.800_ = 1.957,  p = 0.112,  $\eta_{p}^{2}$ = 0.085 |
|  | MMI  N = 12 | 1.318  ± 0.085 | 1.219  ± 0.128 | 1.213  ± 0.129 | 1.254  ± 0.113 | 1.269  ± 0.107 | 1.276  ± 0.098 | 1.251  ± 0.094 |  |  |  |
| POST | UMI  N = 12 | 1.314  ± 0.120 | 1.212  ±0.107 | 1.206  ± 0.110 | 1.269  ±0.107 | 1.269  ±0.106 | 1.277  ± 0.119 | 1.260  ± 0.126 |  |  |  |
|  | MMI  N = 12 | 1.294  ± 0.107 | 1.217  ± 0.117 | 1.218  ± 0.133 | 1.249  ± 0.118 | 1.250  ±0.121 | 1.245  ± 0.126 | 1.230  ± 0.110 |  |  |  |
|  | **Gait Velocity_CoV_ (%)** | | | | | | | | | | |
| PRE | UMI  N = 12 | 17.156  ± 3.759 | 15.439  ± 3.557 | 15.305  ± 4.291 | 15.027  ± 3.513 | 16.269  ± 3.755 | 15.249  ± 3.767 | 15.406  ± 3.823 | F_1,21_ = 0.478  p = 0.497,  $\eta_{p}^{2}$ = 0.022 | F_1,21_ = 0.059,  p = 0.811,  $\eta_{p}^{2}$ = 0.003 | F_3.983,83.646_ = 0.708,  p = 0.589,  $\eta_{p}^{2}$ = 0.033 |
|  | MMI  N = 12 | 16.274  ± 2.375 | 15.734  ± 1.680 | 14.925  ± 1.517 | 14.655  ± 2.330 | 14.757  ± 2.729 | 14.357  ± 2.327 | 14.619  ± 2.412 |  |  |  |
| POST | UMI  N = 12 | 17.017  ± 3.487 | 15.658  ± 3.734 | 15.162  ± 3.867 | 14.835  ± 3.538 | 15.289  ± 3.330 | 16.008  ± 3.094 | 15.837  ± 3.236 |  |  |  |
|  | MMI  N = 12 | 15.711  ± 2.372 | 15.301  ± 2.442 | 14.441  ± 2.467 | 14.714  ± 1.961 | 14.518  ± 2.341 | 14.436  ± 2.107 | 14.917  ± 2.016 |  |  |  |

Table D. Means ± standard deviations for the cognitive and motor dual task costs (DTC) of the pre and post measurement as well as the outcomes of the ANCOVA

| Time | Condition/  Group | Letter Fluency Task  (easy) | Letter Fluency Task  (severe) | Reaction  Time  Task  (easy) | Reaction  Time Task  (severe) | N-Back  Task  (easy) | N-Back  Task  (severe) | DTC Repeated Measures ANCOVA | | |
| --- | --- | --- | --- | --- | --- | --- | --- | --- | --- | --- |
|  |  |  |  |  |  |  |  | ***Time*** | ***Time x Intervention*** | ***Time x Intervention x Condition*** |
|  | **DTC MTC (%)** | | | | | | | | | |
| PRE | UMI  N = 11 | 19.432  ± 8.050 | 22.617  ± 8.101 | 7.350  ± 6.719 | 13.632  ± 13.091 | 10.713  ± 7.322 | 17.324  ± 9.851 | F_1,19_ = 0.0178,  p = 0.678,  $\eta_{p}^{2}$ = 0.009 | F_1,19_ = 1.421,  p = 0.248,  $\eta_{p}^{2}$ = 0.070 | F_3.405,64.694_ = 1.566,  p = 0.201,  $\eta_{p}^{2}$ = 0.076 |
|  | MMI  N = 11 | 15.434  ± 9.710 | 19.054  ± 10.188 | 7.767  ± 7.777 | 8.304  ± 10.701 | 4.017  ± 12.286 | 8.629  ± 15.129 |  |  |  |
| POST | UMI  N = 11 | 20.320  ± 9.836 | 22.613  ± 7.234 | 4.383  ± 8.898 | 8.856  ± 7.671 | 11.004  ± 6.216 | 13.558  ± 8.286 |  |  |  |
|  | MMI  N = 11 | 13.521  ± 11.428 | 16.470  ± 16.045 | 5.886  ± 5.774 | 10.227  ± 5.058 | 9.363  ± 7.036 | 13.225  ± 9.362 |  |  |  |
|  | **DTC MTC_CoV_ (%)** | | | | | | | | | |
| PRE | UMI  N = 11 | -1.717  ± 16.421 | -3.043  ± 11.667 | -3.102  ± 10.660 | 2.553  ± 15.333 | -0.516  ± 9.778 | 2.633  ± 10.442 | F_1,19_ = 0.442,  p = 0.514,  $\eta_{p}^{2}$ = 0.023 | F_1,19_ = 0.052,  p = 0.822, $\eta_{p}^{2}$ = 0.003 | F_2.880,54.727_ = 0.560  p = 0.637 $\eta_{p}^{2}$ = 0.029 |
|  | MMI  N = 11 | -0.627  ±21.677 | 0.011  ± 17.728 | -5.206  ± 15.206 | -6.308  ± 17.811 | -5.103  ± 20.495 | -1.218  ± 16.690 |  |  |  |
| POST | UMI  N = 11 | 2.742  ± 9.703 | -2.410  ± 14.060 | -3.711  ± 19.986 | -1.268  ± 12.177 | 0.831  ± 5.539 | -3.245  ± 15.283 |  |  |  |
|  | MMI  N = 11 | -0.377  ± 18.517 | -5.367  ± 32.634 | -0.330  ± 9.228 | 0.060  ± 7.550 | -7.273  ± 16.044 | -3.097  ± 9.925 |  |  |  |
| **DTC Stride Length (%)** | | | | | | | | | | |
| PRE | UMI  N = 11 | 7.612  ± 4.188 | 8.571  ± 3.488 | 3.320  ± 2.290 | 3.803  ± 3.390 | 4.568  ± 2.829 | 6.227  ± 3.062 | F_1,20_ = 0.000,  p = 0.996,  $\eta_{p}^{2}$ = 0.000 | F_1,20_ = 0.229,  p = 0.638,  $\eta_{p}^{2}$ = 0.011 | F_3.053,61.052_ = 1.594,  p = 0.199,  $\eta_{p}^{2}$ = 0.074 |
|  | MMI  N = 12 | 7.141  ± 6.155 | 7.609  ± 6.134 | 3.571  ± 5.300 | 2.300  ± 6.623 | 1.966  ± 4.324 | 3.922  ± 4.378 |  |  |  |
| POST | UMI  N = 11 | 8.194  ± 3.073 | 8.715  ± 3.177 | 2.523  ± 3.616 | 2.572  ± 3.148 | 3.617  ± 1.207 | 4.978  ± 1.774 |  |  |  |
|  | MMI  N = 12 | 6.164  ± 3.135 | 6.056  ± 5.132 | 3.209  ± 2.806 | 3.120  ± 2.864 | 3.043  ± 3.466 | 4.132  ± 3.876 |  |  |  |
| **DTC Stride Length_CoV_ (%)** | | | | | | | | | | |
| PRE | UMI  N = 11 | -10.420  ± 14.132 | -12.031  ± 17.781 | -10.314  ± 10.793 | -4.151  ± 22.791 | -9.271  ± 8.366 | -9.113  ± 10.680 | F_1,20_ = 0.032,  p = 0.861,  $\eta_{p}^{2}$ = 0.002 | F_1,20_ = 0.259,  p = 0.616,  $\eta_{p}^{2}$ = 0.013 | F_2.997,59.929_ = 2.307,  p = 0.086,  $\eta_{p}^{2}$ = 0.103 |
|  | MMI  N = 12 | -5.935  ± 11.381 | -12.386  ± 9.583 | -17.644  ± 18.892 | -16.856  ± 21.603 | -13.017  ± 16.998 | -12.808  ± 21.986 |  |  |  |
| POST | UMI  N = 11 | -3.888  ± 9.802 | -8.507  ± 12.398 | -13.194  ± 7.302 | -12.231  ± 9.997 | -5.213  ± 5.927 | -7.819  ± 8.992 |  |  |  |
|  | MMI  N = 12 | -7.374  ± 15.207 | -13.996  ± 21.908 | -7.429  ± 13.715 | -8.989  ± 11.464 | -12.778  ± 11.834 | -10.535  ± 12.139 |  |  |  |
| **DTC Gait Velocity (%)** | | | | | | | | | | |
| PRE | UMI  N = 11 | 13.552  ± 7.224 | 15.094  ± 6.141 | 3.418  ± 3.352 | 5.530  ± 8.292 | 5.986  ± 6.045 | 9.543  ± 7.607 | F_1,20_ = 0.006,  p = 0.941,  $\eta_{p}^{2}$ = 0.000 | F_1,20_ = 0.022,  p = 0.883,  $\eta_{p}^{2}$ = 0.001 | F_2.880,57.597_ = 2.128,  p = 0.109,  $\eta_{p}^{2}$ = 0.096 |
|  | MMI  N = 12 | 14.561  ± 13.453 | 16.189  ± 13.629 | 5.345  ± 9.773 | 3.728  ± 11.206 | 2.690  ± 9.069 | 6.055  ± 10.096 |  |  |  |
| POST | UMI  N = 11 | 14.973  ± 6.197 | 16.478  ± 5.930 | 4.194  ± 6.766 | 3.714  ± 6.278 | 4.892  ± 2.515 | 7.769  ± 3.838 |  |  |  |
|  | MMI  N = 12 | 12.187  ± 9.712 | 12.981  ± 12.035 | 4.610  ± 5.618 | 4.632  ± 6.268 | 4.607  ± 7.263 | 6.701  ± 8.480 |  |  |  |
| **DTC Gait Velocity_CoV_ (%)** | | | | | | | | | | |
| PRE | UMI  N = 11 | -8.561  ± 12.638 | -11.004  ± 13.878 | -10.824  ± 9.008 | -4.068  ± 21.083 | -11.065  ± 11.103 | -10.652  ± 13.210 | F_1,20_ = 0.071,  p = 0.792,  $\eta_{p}^{2}$ = 0.004 | F_1,20_ = 2.084,  p = 0.164,  $\eta_{p}^{2}$ = 0.094 | F_3.439,68.786_ = 2.413,  p = 0.066,  $\eta_{p}^{2}$ = 0.108 |
|  | MMI  N = 12 | -5.702  ± 11.172 | -11.065  ± 8.023 | -13.819  ± 13.147 | -13.515  ± 13.402 | -13.520  ± 15.376 | -11.655  ± 16.111 |  |  |  |
| POST | UMI  N = 11 | -4.340  ± 7.690 | -8.418  ± 11.669 | -18.180  ± 10.973 | -13.866  ± 12.671 | -8.774  ± 5.913 | -9.746  ± 10.621 |  |  |  |
|  | MMI  N = 12 | -1.258  ± 10.509 | -7.703  ± 15.195 | -6.252  ± 11.942 | -7.825  ± 9.528 | -11.677  ± 10.494 | -7.820  ± 8.736 |  |  |  |
| **DTC Cognitive Performance (%)** | | | | | | | | | | |
| PRE | UMI  N = 11 | 3.978  ± 21.878 | 2.690  ± 16.599 | 3.164  ± 11.387 | 4.120  ± 7.666 | 1.923  ± 8.737 | -13.791  ± 29.104 | F_1,20_ = 2.384,  p = 0.138,  $\eta_{p}^{2}$ = 0.107 | F_1,20_ = 0.695,  p = 0.414,  $\eta_{p}^{2}$ = 0.034 | F_2.459,49.182_ = 0.936,  p = 0.415,  $\eta_{p}^{2}$ = 0.045 |
|  | MMI  N = 12 | 3.875  ± 20.612 | -5.161  ± 22.839 | 4.132  ± 10.399 | 5.605  ± 8.161 | 2.111  ± 7.450 | 3.691  ± 13.273 |  |  |  |
| POST | UMI  N = 11 | 3.151  ± 12.696 | 8.043  ± 15.231 | 6.090  ± 6.517 | 9.671  ± 6.416 | -4.437  ± 9.525 | -4.462  ± 20.288 |  |  |  |
|  | MMI  N = 12 | 4.765  ± 15.189 | 5.742  ± 23.958 | 5.279  ± 7.211 | 5.473  ± 6.374 | -1.591  ± 4.473 | -3.420  ± 19.539 |  |  |  |

Table E. Means ± standard deviations for the cognitive performance measures performed during the pre and post measurements as well as the outcomes of the ANCOVA

| Time | Condition/ Intervention | Single-Task | | | | | | | Dual-Task | | | | | |
| --- | --- | --- | --- | --- | --- | --- | --- | --- | --- | --- | --- | --- | --- | --- |
|  |  | Letter Fluency Task (n)  (easy) | Letter Fluency Task (n)  (severe) | Reaction  Time  Task (s)  (easy) | | Reaction Time  Task (s)  (severe) | N-Back  Task  (n)  (easy) | N-Back  Task  (n)  (severe) | Letter Fluency Task  (n)  (easy) | Letter Fluency Task  (n)  (severe) | Reaction  Time  Task  (s)  (easy) | Reaction  Time  Task  (s)  (severe) | N-Back  Task  (n)  (easy) | N-Back  Task  (n)  (severe) |
| PRE | UMI  N = 11 | 3.546  ± 11.588 | 26.636  ± 10.557 | 0.928  ± 0.149 | | 0.868  ± 0.129 | 29.273  ± 1.104 | 21.818  ± 5.741 | 32.723  ± 10.527 | 25.727  ± 10.412 | 0.966  ±0.160 | 0.907  ± 0.124 | 28.636  ± 1.748 | 22.091  ± 6.580 |
|  | MMI  N = 12 | 37.083  ± 11.024 | 31.000  ± 10.100 | 0.979  ± 0.170 | | 0.908  ± 0.124 | 29.500  ± 1.446 | 23.500  ± 3.920 | 34.750  ± 9.836 | 31.417  ± 8.554 | 1.019  ± 0.121 | 0.963  ± 0.114 | 28.833  ± 2.082 | 22.417  ± 3.729 |
| POST | UMI  N = 11 | 37.636  ± 11.474 | 31.091  ± 7.867 | 0.873  ± 0.189 | | 0.794  ± 0.147 | 27.909  ± 3.390 | 23.091  ± 5.029 | 36.091  ± 10.212 | 28.181  ± 7.494 | 0.932  ± 0.202 | 0.886  ± 0.194 | 29.000  ± 3.000 | 23.636  ± 4.632 |
|  | MMI  N = 12 | 42.583  ± 8.152 | 35.917  ± 9.434 | 0.931  ± 0.238 | | 0.885  ± 0.242 | 29.417  ± 1.379 | 25.167  ± 3.589 | 40.583  ± 10.013 | 32.917  ± 8.795 | 0.984  ± 0.232 | 0.931  ± 0.229 | 29.833  ± 0.577 | 25.500  ± 2.505 |
| Repeated Measures ANCOVA | | | | | | | | | | | | | | |
| ***Time*** | | | | | ***Time x Intervention*** | | | | | | ***Time x Intervention x Condition*** | | | |
| F_1,20_ = 0.019,  p = 0.892, $\eta_{p}^{2}$ = 0.001 | | | | | F_1,20_ = 0.532  p = 0.474, $\eta_{p}^{2}$ = 0.026 | | | | | | F_4.585,97.162_ = 0.249  p = 0.936, $\eta_{p}^{2}$ = 0.012 | | | |
